# Assessing Adolescent Functioning from Different Perspectives: Extending the Validation of the Adolescent Functioning Scale (AFS)

**DOI:** 10.1007/s10578-022-01428-2

**Published:** 2022-09-09

**Authors:** Cassandra K. Dittman, Kylie Burke, Divna Haslam, Alan Ralph

**Affiliations:** 1 Central Queensland University, Locked Bag 3333, Bundaberg DC, QLD 4670 Australia; 2 Parenting and Family Support Centre, School of Psychology, The University of Queensland, Brisbane, Australia; 3 Metro North Health Service (Mental Health), Queensland Health, Brisbane, Australia; 4 Queensland University of Technology, Brisbane, Australia; 5 Triple P International, Brisbane, Australia

**Keywords:** Adolescent, Parent, Questionnaire, Validation, Problem behavior, Emotional difficulties, Positive development

## Abstract

The study aimed to provide further evidence for the validity of the 33-item Adolescent Functioning Scale (AFS) as a parent- and adolescent-report scale of adolescent adjustment. In separate samples of parents (*N* = 542; 88% female) and adolescents (*N* = 303; 60% female), confirmatory factor analyses supported the original 4-factor structure of the AFS. Analyses produced a 28-item parent measure, and a 27-item adolescent measure. Parent and adolescent versions included positive development, oppositional behaviour, antisocial behaviour and emotional problems subscales. Evidence for convergent and construct validity was provided through correlations with existing measures of adolescent functioning and parenting. The AFS demonstrated configural and metric invariance, but not scalar variance. The study provided support for the validity and reliability of the revised AFS for parents and adolescents. The strong psychometric properties, and brief and multi-dimensional nature of the AFS means that it will have utility in research and applied contexts.

**Supplementary Information:**

The online version contains supplementary material available at 10.1007/s10578-022-01428-2.

## Introduction

Adolescence is a dynamic developmental period. Adolescents experience important improvements in cognitive and self-regulatory capacity, transitions in the nature of peer and intimate relationships, increasing autonomy from parents and family, and a growing sense of identity and personal agency [1]. For most, these experiences result in young people who are future-oriented, and motivated to become contributing and connected community members [2]. On the other hand, adolescence is also a period of vulnerability for mental health disorders. The evidence is compelling: mental health disorders are the main cause of worldwide disability among 10–24-year-olds [3]; 75% of mental disorders are present before the age of 24 years and 50% before 14 years [4]; and engagement in risky behavior in adolescence is associated with adverse health, social and employment outcomes in early adulthood [5]. National and international policy now acknowledges that comprehensive strategies supporting adolescent wellbeing are critical components for protecting the mental health of communities across the life course [1, 6, 7].

Successful mental health strategies require effective mechanisms for assessing outcomes. There is a growing push for outcome measures to be routinely incorporated into service implementation for quality assurance, service evaluation, and to track risk factors and mental health trends across time [8, 9]. Previous literature has however, reported that the degree of concordance between parent-proxy and adolescent self-reports of adolescent functioning varies across behaviors [10]. The consensus therefore is that multi-informant, multi-method assessments, including both parent-proxy and adolescent self-report, provide the most valid way of assessing adolescent functioning in clinical and community samples [10–13]. Currently, few measures of adolescent adjustment allow multiple perspectives to be examined in a way that possesses both psychometric credibility and utility in practice and research [14]. This paper describes further validation of the Adolescent Functioning Scale [AFS; 15] to provide parallel forms of the measure for parents and adolescents.

The initial AFS study was the first step in the development and validation of a new, brief measure of adolescent functioning [15]. This addressed several problems with existing measures highlighted in recent systematic reviews of measures for adolescents [14, 16] including: a lack of differentiation between developmental phases of childhood and adolescence; too little emphasis on assessing a broad range of developmental assets and competencies; and limited accessibility (e.g., fees for use or restricted to certain professionals). Thus, the aim of test construction was to develop a scale that sampled items and assessed multiple domains of functioning relevant to the adolescent period, including positive development and behavioral and emotional problems. Further, the scale was designed to track intervention outcomes in prevention, early intervention and treatment contexts; be suitable for use in research, clinical and community settings; and possess cross-cultural applicability.

This first study provided evidence for the psychometric properties of the subscale scores with a sample of 278 parents of adolescents aged 11–18 years [15]. The 33-item measure of adolescent functioning comprised four subscales assessing oppositional defiant behavior, antisocial behavior, emotional difficulties, and positive development. The results provided evidence for strong internal and test–retest reliability, change sensitivity, and support for content and construct (convergent and discriminant) validity. This paper reports the findings from the next phase in construction of the AFS, which focuses on developing a parallel version of the scale for completion by adolescents.

## Aims and Hypotheses

The study employed separate samples of parents and adolescents and aimed to: (1) further establish the construct validity of the interpretation of AFS scores with a new sample of parents via confirmatory factor analysis (CFA); (2) provide evidence for the validity of the interpretation of AFS scores for use by adolescents (11–17 years); and (3) assess equivalence of the factor structure and interpretation of the AFS scores across the parallel parent and adolescent forms via invariance testing. We hypothesised that the: (1) 4-factor structure for the parent-report AFS would be confirmed with a new sample of parents; (2) same 4-factor structure would apply for the adolescent-report version; (3) 4-factor structure of the AFS would show measurement invariance across the parent and adolescent forms; and (4) parallel versions would show an expected pattern of correlations across existing measures of adolescent functioning and parenting.

## Method

### Participants

#### Parent Sample

Participants were 542 parents of young people aged 11–17 years. Parents were recruited via community and online outreach, particularly via paid and shared posts on Facebook and through advertising in newsletters in secondary schools. Parents ranged in age from 27 (this parent was a stepmother to an 11-year-old) to 72 years (a grandmother in a primary caregiver role; *M* = 44.44 years; *SD* = 6.52). Target adolescents’ mean age was 14.11 years (*SD* = 1.77). Most respondents were mothers (88%) who identified as Australian (69%), although European (15%), New Zealander (6%) and Asian (5%) backgrounds were represented. Seven percent of parents had not completed secondary school, 14% had completed secondary school, 23% had a trade or technical/vocational college qualification, and 56% had a University education. Most adolescents lived with both biological parents (65%), 19% lived in sole-parent families and 16% in step- or blended families.

#### Adolescent Sample

This sample was drawn from adolescents taking part in online survey studies (*n* = 245, 81%) or randomised controlled trials (RCT) of parenting interventions (*n* = 58, 19%) conducted by our research group. The recruitment processes outlined above for the parent sample applied to the adolescent sample, as adolescents were recruited via parent invitation into the study. The invitation and consent process involved two steps. For online responses, parents were asked if they consented for their nominated adolescent to be contacted to take part in a parallel survey. Parents who consented provided their adolescent’s email address, and the adolescent was sent a separate link to the online survey. Adolescents gave online consent prior to accessing the survey. When parents completed a hard copy survey, they gave signed written consent for their adolescent, and then gave their adolescent their own consent form and survey booklet. Adolescents gave signed written consent and returned their consent form and survey in a separate envelope.

The sample of 303 adolescents was aged between 11 and 17 years (*M* = 14.14 years, *SD* = 1.94). Most identified as female (60% vs 39% male) and < 2% identified as being neither male nor female. All adolescents were engaged in education with 5% currently attending primary school, 72% attending secondary school and 22% attending University.^1^ Most adolescents identified as Australian (72%) and 3% reported an Australian First Nation cultural background. There was representation from Asian (6%) and European (3%) backgrounds. When reporting on parenting, most adolescents reported on their mother (71%), as this was the parent who had invited them into the study. The highest level of education for that target parent was reported by adolescents as 11% having not completed secondary school, 15% with a secondary school education, 14% with a trade or vocational college qualification and 41% with a university-level education. Nineteen percent of adolescents did not know their parent’s education level. A small sub-set of adolescents (*n* = 30) from the RCT had corresponding parent data, meaning that there were 30 parent-adolescent dyads in this study.

### Procedure

Ethics approval for all studies was obtained from the authors’ University Human Research Ethics Committee.^2^ All parents provided informed consent (either written or online). Both parental and adolescent consent/assent was obtained for adolescent participants (see above). This study was not preregistered.

Please see Authors et al. (year) for full details of the test construction process used for the initial parent-report AFS. The 33-item AFS was included in a questionnaire battery. Participants rated each statement on a 6-point Likert scale ranging from 0 (*not at all true*) to 5 (*nearly always or always true*) regarding their own or their adolescent’s functioning over the past 4 weeks*.* Parent items were re-worded into the first person for completion by adolescents (e.g., *My teenager constantly seeks reassurance* became *I constantly seek reassurance*). The survey also contained demographic items (i.e., parent age, gender, educational level, cultural background; family structure; and adolescent age and gender) and the measures to provide evidence for the validity of the AFS test score interpretations. Alphas reported below are based on the current parent and adolescent samples.

### Validation Measures

#### Adolescent Functioning

Parents and adolescents completed the Youth Outcome Questionnaire [YOQ; 17] to obtain evidence for the construct (convergent) validity of the interpretations of AFS test scores. The YOQ was designed to track psychotherapy outcomes in clinical populations. Three of the six YOQ subscales were used. The 10-item Interpersonal Relations scale assessed disruptive and oppositional behavior applicable to relationships with others (e.g., aggressiveness, defiance, arguing; parent α = 0.87; adolescent α = 0.82). The 8-item Social Problems subscale assessed antisocial behavior (e.g., truancy, drug/alcohol use, rule violations; parent α = 0.82; adolescent α = 0.74). Finally, the 18-item Intrapersonal Distress subscale assessed emotional symptoms (e.g., sadness, reduced pleasure in activities, anxiety; parent α = 0.94; adolescent α = 0.93). Adolescents rated each item as to how true it was in the last 7 days on a 5-point scale from 0 (*never or almost never*) to 4 (*always or almost always*).

#### Parenting

Parents and adolescents completed the 9-item, short form of the Alabama Parenting Questionnaire [APQ; 18] to assess parenting practices. Based on the original 42-item APQ [19], the short form comprises 3 subscales each containing 3 items that describe a parenting practice. The APQ measured Positive Parenting (parent α = 0.85; adolescent α = 0.86), Inconsistent Discipline (parent α = 0.75; adolescent α = 0.68) and Poor Supervision (parent α = 0.82; adolescent α = 0.77). Parents rated how often they typically used each practice on a 5-item scale from 1 (*never*) to 5 (*always*), while adolescents rated how often they experienced each practice with their nominated parent (i.e., the parent who consented for them to take part in the study).

#### Parent-Adolescent Relationship

Parents and adolescents also completed the 15-item Parent-Adolescent Relationship Scale [PARS; 20]. The questionnaire assessed the parent-adolescent relationship across three subscales: Connectedness (warmth and support; parent α = 0.88; adolescent α = 0.90); Shared Activities (spending time in mutually enjoyable activities; parent α = 0.73; adolescent α = 0.73); and Hostility (negativity and criticism; parent α = 0.78; adolescent α = 0.72). Participants rated each statement on a scale from 0 (*not at all true*) to 5 (*nearly always or always true*) about the relationship with their parent or adolescent.

### Data Analysis

The parent sample was used to conduct Confirmatory Factor Analysis (CFA) to confirm the structure of the parent version, further refine the number of items on the measure and verify the scale’s internal consistency and construct validity. The final parent version was then evaluated with the adolescent sample. CFA was used to confirm the factor structure of the adolescent version and invariance testing was used to test for equivalence across the parent and adolescent versions. Construct validity of the interpretation of scores on the final versions of the parent and adolescent versions of the AFS was examined through correlations with conceptually related existing measures. Data from this study is available through written request to the corresponding author.

#### Confirmatory Factor Analysis

AMOS Version 27 was used to conduct CFA. First, the factor structure was confirmed for the parent version by one-factor congeneric models (in which the relationship between indicators and the latent variable are direct) for each of the four subscales: Positive Development, Oppositional Defiant Behavior, Emotional Difficulties and Antisocial Behavior. This step assessed that the factors were unidimensional and that the expected indicator variables contributed to the overall latent variable of the theorised constructs. Each model was respecified (if indicated) via the removal of items demonstrating high levels of shared variance on the Standardised Residuals or Modification Indices and according to theoretical coherence with the construct. Items considered less reflective of the subscale construct or to have higher complexity were then removed. Then, the measurement models for the four AFS subscales were combined in a 4-factor model to assess whether the subscales each measured a unique aspect of adolescent functioning (i.e., discriminant validity within the AFS). The 4-factor model was then run with the adolescent sample.

Four statistical criteria [21, 22] were used to guide assessment of model fit: non-significant Chi-square statistic (*p* > 0.05); Standardised Root Mean Square Residual (SRMR; < 0.08); Comparative Fit Index (CFI: acceptable ≥ 0.90; superior ≥ 0.95); and the Root Mean Square Error of Approximation (RMSEA; close fit < 0.05; acceptable fit < 0.08). The Antisocial Behavior subscale of the parent and adolescent versions violated assumptions of multivariate normality using Mardia’s Coefficient. The Bollen-Stine *p* bootstrapping procedure was therefore completed as a post-hoc adjustment to allow for non-normality of the data [23, 24]. A non-significant Bollen-Stine *p* is indicative of good fit [23].

#### Invariance Testing

Multi-group CFAs were conducted using a structural equation modelling (SEM) framework to assess measurement invariance for the parent and adolescent versions. A hierarchical series of tests was employed to sequentially assess the three aspects of measurement invariance that are required for comparing latent means across groups: configural, metric and scalar.

Configural invariance was assessed to establish the degree to which the constructs have the same basic organisation across models (the items loaded on the specified factors across the groups). A multi-group model was specified to simultaneously assess the factor structure across the groups. The indicators with the smallest differences across the groups for each factor were set to unity. If configural invariance was supported, this implied that the 4-factor structure of the AFS is supported (although not necessarily equivalent) across parent and adolescent versions.

Metric invariance was tested next to establish if there was equivalence of factor loadings between groups, such that each item contributes to the latent construct to a similar degree across groups [25]. A model with factor loadings constrained to be equivalent across groups was compared to the configural model. If overall model fit of the metric invariance model was significantly worse, at least one of the factor loadings was not equivalent across groups, and metric invariance was not supported. The final step was to determine scalar invariance to assess whether mean differences in the latent constructs captured all mean differences in the shared variance of the items [25]. Constraints were imposed on factor loadings and item intercepts.

At each test, if invariance was unsupported, each factor loading (metric) and intercept (scalar) was individually assessed for non-invariance with non-invariant factor loadings or intercepts, then allowed to freely estimate and the model rerun. If more than 50% of items were invariant, then the model was rejected and no further testing was conducted. Metric and scalar invariance were assessed using ∆χ^2^ with *p* > 0.05 supporting invariance. However, ∆χ^2^ is sensitive to sample size, so Chen’s criteria [26] was applied in the present study: a − 0.01 change in CFI for nested models paired with changes in RMSEA (0.15) and SRMR (metric: 0.030; scalar: 0.015).

## Results

### Confirming the Parent-Report AFS

Table 1 provides goodness of fit statistics for each factor and the final model. Table 2 lists the items, standardised factor loadings and items removed from the four subscales.

**Table 1:** Final adolescent functioning scale CFA models: goodness-of-fit statistics for parent and adolescent versions

Model	χ2	df	*p*	CFI	RMSEA (CI)	SRMR	Bollen-Stine *p*
Initial parent 4-factor model, 33 items (*N* = 542)	1924.82	489	.000	.85	.07 (.07, .08)	.08	
Parent 4-factor model, 28 items (*N* = 542)	1056.01	343	.000	.90	.06 (.06, .07)	.07	–
Positive development, 9 items	102.84	27	.000	.95	.07 (.06, .09)	.04	^a^
Oppositional defiant behavior, 8 items	83.36	20	.000	.98	.08 (.06, .09)	.03	^a^
Emotional difficulties, 5 items	11.14	4	.025	.99	.06 (.02, .11)	.02	^a^
Antisocial behavior, 6 items	42.66	9	.000	.98	.08 (.06, .11)	.03	.074
Adolescent 4-factor, 27 items (*N* = 303)	674.46	316	.000	.86	.06 (.06, .07)	.07	–
Positive development, 9 items	59.82	27	.000	.94	.06 (.04, .09)	.05	^a^
Oppositional defiant behavior, 8 items	64.25	20	.000	.94	.09 (.06, .11)	.05	^a^
Emotional difficulties, 5 items	2.67	4	.615	1.00	.00 (.00, .07)	.01	^a^
Antisocial behavior, 5 items	8.73	4	.068	.99	.06 (.00, .12)	.03	.103

*CFI* Comparative fit index, *RMSEA* Root mean square error of approximation, *CI* Confidence interval, *SRMR* Standardized root mean square residual

^a^Normally distributed at .01 level, therefore no Bollen-Stine *p* to report

**Table 2:** Items and standardized regression weights for the final parent and adolescent versions by subscale

No	Item	Standardized factor loading		
		Initial 33-item parent version	28-item Parent version	27-item Adolescent version
Positive development				
6	Gets involved in activities at school or in the community	.57	.57	.47
8	Talks about their views, ideas and needs appropriately	.56	.56	.61
10	Is good at planning ahead for big tasks (e.g. assignments or exams)	.74	.74	.72
17	Tries hard at school/work/university	.75	.75	.68
19	Doesn’t give up after a setback	.62	.61	.58
21	Asks for advice about serious issues (e.g. drugs, sex, or relationships) ^b^	.44	.44	.33
23	Does things for themselves	.51	.51	.36
28	Thinks through consequences before acting	.69	.69	.52
30	Has goals for the future	.66	.66	.58
Oppositional defiant behavior				
2	Hurts me or others (e.g. hits, pushes, kicks)	.59	.56	.39
3	Over-reacts	.80	–	–
4	Yells, shouts or screams^b^	.84	–	–
5	Loses their temper	.85	.79	.63
7	Argues or fights with their brothers or sisters^a^	.54	–	–
15	Rudely answers back to me	.84	.85	.76
16	Refuses to do jobs around the house when asked ^b^	.68	.70	.62
18	Is irritable	.74	.75	.51
25	Threatens others^b^	.67	–	–
26	Gets upset or angry when they don’t get their own way	.81	.82	.69
27	Whines or complains	.79	.81	.67
29	Talks back or argues when asked to do something	.78	.82	.79
Emotional difficulties				
1	Constantly seeks reassurance	.49	.53	.44
11	Puts themselves down	.71	.68	.72
14	Seems unhappy or sad	.75	.69	.70
31	Seems fearful and scared	.69	.73	.75
32	Worries	.68	.72	.74
24	Seems to feel good about themselves^b^	.60	–	–
Antisocial behavior				
9	Gets into trouble at school/college/work^b^	.64	.64	–
12	Uses tobacco, drugs or alcohol	.73	.73	.67
13	Comes home late or misses their set curfew	.75	.75	.76
20	Engages in risky or unhealthy activities	.82	.82	.60
22	Skips school, classes or work	.75	.75	.71
33	Spends time with undesirable peers	.70	.70	.44

^a^Item was removed prior to analysis

^b^Item removed during one-factor congeneric testing

^c^Items are worded as per the parent-report version

As the intention of the study was to develop a measure with parallel parent and adolescent versions, the first step in confirming the parent version was a conceptual review of items to ensure relevance and generalisability of items across parents and adolescents and family types. This review resulted in the removal of item 7 as it referred to siblings, which would limit applicability for sole child families. This resulted in a 32-item version for analysis. However, initial 33-item fit statistics and factor loadings have been included in Tables 1 and 2 for reference.

#### One-Factor Congeneric Models

##### Positive Development

The one-factor congeneric model of Positive Development revealed that the nine items were not a good fit for the hypothesised factor (χ^2^ = 102.84, *df* = 27, *p* < 0.000). However, approximate fit indices provided support for model fit [CFI = 0.95; RMSEA = 0.07 (0.06, 0.09); SRMR = 0.04]. Standardised factor loadings were all significant (*p* < 0.001 level), ranging from 0.45 to 0.74. Inspection of standardised residual covariances and modification indices indicated issues with shared variance for item 21. However, re-specification did not result in meaningful improvements to the model fit and so the 9-item model was retained (see Table 2).

##### Oppositional Defiant Behavior

The one-factor congeneric model of the 11-item Oppositional Defiant Behavior scale revealed the data was not a good fit for the hypothesised factor [χ^2^ = 462.08, *df* = 44, *p* < 0.001; CFI = 0.90; RMSEA = 0.13 (0.12, 0.14); SRMR = 0.05]. The standardised factor loadings were all significant at the *p* < 0.001 level, ranging from 0.59 to 0.85. In addition to item 7, which had previously been removed, re-specification led to removal of 3 items: 3, 4 and 25. This resulted in support for fit for the model (see Table 1) and this 8-item model was retained.

##### Emotional Difficulties

The one-factor congeneric model of the 6-item Emotional Difficulties construct revealed that the data was not a good fit for the hypothesised factor [χ^2^ = 83.30, *df* = 9, *p* < 0.001; CFI = 0.93; RMSEA = 0.12 (0.10, 0.15); SRMR = 0.05]. The standardized factor loadings were all significant at *p* < 0.001, ranging from 0.49 to 0.73. Re-specification resulted in the removal of item 24 and covarying of items 11 and 14. This resulted in data having reasonable fit for the model (see Table 1) and hence the 5-item model was retained.

##### Antisocial Behavior

The one-factor congeneric model of the 6-item Antisocial Behavior construct revealed that the data was not a good fit for the hypothesised factor (χ2 = 42.66, *df* = 9, *p* < 0.001). However, comparative fit indices provided support for the model [CFI = 0.98; RMSEA = 0.08 (0.06, 0.11); SRMR = 0.03] with the Bollen-Stine *p* non-significant (*p* = 0.07). The standardized factor loadings were all significant at *p* < 0.001, ranging from 0.61 to 0.82. Thus, the final Antisocial Behavior model retained all six items.

To assess whether the four factors had adequate discriminant validity and measured a unique element of the adolescent functioning, a 4-factor independent-cluster model was specified; the factor intercorrelations were freely estimated. The data did not fit the model well. However, this was expected given the number of indicators and lack of normality. Comparative fit indices provided support for the model. Standardised factor loadings were all significant and, except for item 21, were above 0.50 (see Table 2). Correlations between factors were in the expected direction: Emotional Difficulties to Positive Development, *r* = − 0.31; Antisocial to Emotional Difficulties, *r* = 0.37; Antisocial to Positive Development,* r* = − 0.44; Positive Development to Oppositional Defiant Behavior,* r* = − 0.44; Oppositional Defiant Behavior to Antisocial, *r* = 0.49; Oppositional Defiant Behavior to Emotional Difficulties,* r* = 0.60. To further assess discriminant validity, a chi-square test of independence was performed to check if the observed chi-square would differ significantly if the correlation between the largest correlation between the four factors, ODB and ED (*r* = 0.60), was forced to be 1. Results indicated a significant deterioration in fit, with chi-square increasing by 24.75 points and 1 degree of freedom to χ2 = 1080.77, *df* = 344, *p* < 0.001. Thus, this finding supported discriminant validity, indicating that each of the factors contributed unique variability to the measurement of adolescent functioning. The 28-item, 4-factor model was retained as the final AFS parent-report version.

### Confirming the Adolescent-Report Version of the AFS

#### One-Factor Congeneric Models

The next step was to determine whether the 28-item, 4-factor structure of the AFS fitted the adolescent data by assessing one-factor congeneric models for each subscale and then testing the multi-factor measurement model. Results are presented in Tables 1, 2, 3 and provided support for the 4-factor model but with 27 items rather than 28. One-factor congeneric models for the Positive Development and Emotional Difficulties subscales adequately fit the data with no further re-specifications required. The initial results for the Oppositional Defiant Behavior subscale also provided some support for the model with no justifiable re-specifications. However, the one-factor congeneric for the Antisocial subscale indicated that, while there was some support for this subscale via the CFI and SRMR, other fit indices did not reach the recommended thresholds [χ2 = 48.88, *df* = 9, *p* < 0.001; CFI = 0.91; RMSEA = 0.12 (0.09, 0.16); SRMR = 0.06; Bollen Stine *p* = 0.001]. Inspection of the Standardised Regression Weights revealed that item 9 had a relatively low loading (0.37) and the Standardised Residuals and Modification Indices indicated the model would be improved by covarying items 9 and 20 and items 33 and 20. Given the low loading for item 9, suggesting this item was contributing relatively little to the construct, the item was removed and the model rerun with items 33 and item 20 allowed to covary. Results provided support for a modified 5-item model (see Table 1) and as such, this model was retained.

**Table 3:** Testing for configural, metric, scalar and residual measurement invariance for parent and adolescent versions

Model	χ^2^	df	CFI	Model Comp	RMSEA (CI)	SRMR	∆χ^2^ (df)	∆CFI	∆SRMR	∆RMSEA	Decision
Parent versus adolescent model equivalence (allowing models to differ)											
M1: Configural	1730.472**	659	.892		.044 (.04, .05)	.0676	–	–	–	–	–
M2: Metric	1770.634**	682	.890	M1	.044 (.04, .05)	.0695	40.162 (23)*	.002	− 0.002	− 0.000	Accept
M3: Scalar	2021.386**	709	.867	M2	.047 (.04, .05)	.0699	250.752 (27)**	.023	− 0.004	− 0.003	Reject

^a^Criteria: ∆CFI = − .01; ∆SRMR = .030 (metric); .015 (scalar); ∆RMSEA = 0.15

*p < .05

**p < .001

Specification and evaluation of a fully independent cluster 4-factor measurement model in which the factor inter-correlations were freely estimated using the Positive Development, Emotional Difficulties, Oppositional Defiant Behavior and modified one-factor congeneric model for Antisocial Behavior showed that the data did not fit the model well. However, comparative fit indices, RMSEA and SRMR, provided some support for the model with the CFI almost reaching threshold. The standardised factor loadings were significant, ranging from 0.32 to 0.72. Despite CFI not quite achieving the recommended threshold, inspection of Modification Indices did not indicate any justifiable changes. As we aimed to test whether equivalent parallel measures for parents and adolescents were feasible, it was decided to proceed to invariance testing using the 27-item adolescent model.

Correlations between factors were small to moderate and in the expected direction, ranging from Antisocial to Positive Development, *r* = − 0.06; Emotional Difficulties to Positive Development, *r* = − 0.11, Oppositional Defiant Behavior to Positive Development, *r* = − 0.23; Antisocial to Emotional Difficulties,* r* = 0.39; Oppositional Defiant Behavior to Antisocial,* r* = 0.49; and. Oppositional Defiant Behavior and Emotional Difficulties, *r* = 52. To further check discriminant validity between the AFS subscales, chi-square tests of independence were performed to assess for significant reductions in model fit if the largest correlation between the four factors, Oppositional Defiant Behavior and Emotional Difficulties (*r* = 0.52) was forced to be 1. Results demonstrated a significant deterioration in fit; the chi-square for the adolescent model increased by 41.30 points and 1 degree of freedom to χ^2^ = 715.76, *df* = 317, *p* < 0.001. These results support discriminant validity, indicating that each of the factors made a unique contribution to the measurement of adolescent functioning.

### Testing Equivalence of the Parent- and Adolescent-Report Versions

The next step in assessing the structure and equivalence of the adolescent-report version was to test measurement invariance to assess if the latent variables and indicators were equivalent between the adolescent- and parent-report versions. This step establishes whether the constructs have similar or different meanings depending on who the respondent is and has implications for whether comparisons across means are valid. As there were minor differences between the parent and adolescent models, invariance tests were run allowing for the paths to differ (see Table 3).

Firstly, configural invariance was assessed by setting the indicator with the smallest difference across groups to unity for each factor and then running the unconstrained model to simultaneously assess the factorial structure across groups. Configural and metric invariance were supported. However, scalar invariance was not initially supported. Tests of each individual intercept revealed that 70% of items were non-invariant and scalar invariance could not be supported (see Table 3). Overall, the results supported configural and metric invariance, providing support for the 4-factor model and equivalence of factor loadings between groups. However, lack of support for scalar invariance indicated that the mean differences in the latent constructs did not capture all mean differences in the shared variance of items. Thus, the means between groups are likely to differ.

### Psychometric Properties of the Final 28-item AFS Parent Version and 27-item Adolescent Version

#### Internal Consistency and Validity

CFA confirmed the 4-factor structure of the AFS with some refinement of items to produce a measure that fit well across two parallel report forms: parent and adolescent. While the 4-factor structure of the AFS was consistent across the parent and adolescent versions, the final scales differed slightly. The parent AFS contained 28 items and the adolescent AFS, 27 items. Three subscales: Positive Development (9 items), Oppositional Defiant Behavior (8 items) and Emotional Difficulties (5 items) were consistent between groups. The Antisocial Behavior subscale had one less item for adolescents after the removal of item 9. Based on this final model, subscales scores were created by summing relevant items and dividing by the total number of items.

See Table 4 for *H*-coefficients, descriptive statistics and correlations with other measures. Internal consistency for all subscales and versions was excellent. Evidence for the convergent validity of the interpretation of AFS test scores on the parent and adolescent versions was provided through moderate to large correlations in the expected directions with existing measures of adolescent oppositional (YOQ-Interpersonal Relations), antisocial (YOQ-Social Problems) and emotional functioning (YOQ-Intrapersonal Distress). The AFS Positive Development subscale was negatively correlated with each of the subscales on the YOQ, except for the correlation with YOQ-Social Problems. Similarly, the AFS subscale scores were correlated in expected ways with measures of factors known to influence or contribute to adolescent functioning. Specifically, the AFS subscale scores were associated with measures of effective (APQ-Positive Parenting) and ineffective parenting (APQ-Poor Supervision, APQ-Inconsistent Discipline), and with positive (PARS-Connectedness, PARS-Shared Activities) and negative dimensions (PARS-Hostility) of the parent-adolescent relationship. Investigation of concordance with a small sub-sample of 30 parent-adolescent dyads indicated high concordance between parent and adolescent scores on all four subscales: Positive Development (*r* = 0.77, *p* < 0.001), Oppositional Defiant Behavior (*r* = 0.84, *p* < 0.001), Emotional Difficulties (*r* = 0.71, *p* < 0.001) and Antisocial Behavior (*r* = 0.91, *p* < 0.001).

**Table 4:** Final parent and adolescent versions of the adolescent functioning scale: descriptive statistics, internal consistency and evidence for convergent validity

	Parent (*N* = 542)				Adolescent (*N* = 303)			
	Positive development	Oppositional defiant behavior	Emotional difficulties	Antisocial behavior	Positive development	Oppositional defiant behavior	Emotional difficulties	Antisocial behavior
Mean (*SD*)	3.15 (1.03)	1.52 (1.11)	1.48 (1.03)	0.43 (0.75)	3.09 (0.94)	1.39 (0.93)	1.84 (1.18)	.68 (.88)
*H*-Coefficient	.84	.93	.81	.88	.78	.87	.83	.80
Adolescent functioning								
YOQ interpersonal relations	− .53***	.80***	.54***	.65***	− .31***	.55***	.38***	.42***
YOQ social problems	-.33***	.63***	.39***	.83***	− .13	.49***	.27**	.74***
YOQ intrapersonal distress	− .41***	.66***	.77***	.53***	− .33***	.58***	.76***	.26***
Parenting								
APQ positive parenting	.28***	− .24***	− .19***	–. 12*	.39***	− .19**	− .20***	− .09
APQ poor supervision	− .29***	.43***	.23***	.75***	− .16**	.30***	.16**	.66***
APQ inconsistent discipline	− .17**	.41***	.22***	.25***	− .15*	.34***	.05	.28***
Parent-adolescent relationship								
PARS connectedness	.36***	− .27***	− .14**	− .20***	.45***	− .21***	− .20***	− .13*
PARS shared activities	.32***	− .23***	− .07	− .21***	.38***	− .22***	− .25***	− .17**
PARS hostility	− .41***	.68***	.38***	.47***	− .26***	.52***	.37***	.23***

*YOQ* Youth outcome questionnaire (Parent *N* = 166; Adolescent *N* = 138), *APQ* Alabama parenting questionnaire (Parent *N* = 324; Adolescent *N* = 291; *PARS* parent-adolescent scale (Parent *N* = 540; Adolescent *N* = 300)

*p < .05

**p < .01

***p < .001

## Discussion

The study aimed to build on the original validation of the Adolescent Functioning Scale [AFS; 15] by providing further validation and refinement of the parent-report measure in a separate parent sample, alongside the validation of an adolescent-report version in a sample of adolescents aged 11–17 years. Results provided support for the utility of the AFS as a multi-dimensional, multi-informant instrument to assess positive development and problem behavior in adolescents from both parent and adolescent perspectives. Consistent with the initial psychometric evaluation [15], the current study indicated that the AFS has good internal consistency, with high *H* coefficients for all subscales and across both versions. Further, the study provided evidence for the convergent validity of the interpretation of the AFS subscale scores, as it correlated in expected ways with relevant scales on a clinical measure of adolescent mental health (YOQ), and with measures of parenting (APQ) and the parent-adolescent relationship (PARS).

Confirmatory factor analyses provided additional evidence for the construct validity of the interpretation of the AFS subscale scores. This study confirmed that the factor structure for the identified subscales, Positive Development, Oppositional Defiant Behavior, Antisocial Behaviour, Emotional Difficulties, holds across a second sample of parents, and across both adolescent and parent versions. These analyses allowed for refinement of the scale, reducing the initial 33-item parent version to 28 items for parents and 27 items for adolescents. This reduction in items provides time and participant burden advantages. Correlations between the AFS subscales were not sufficiently high to recommend the use of a total scale score comprising the sum of all items. This is conceptually logical given the AFS includes both positive and negative dimensions of adolescent functioning. Thus, the subscales of the AFS function as measures of distinct yet related constructs that are best considered alongside one another to provide a comprehensive profile of an adolescent’s functioning across multiple domains.

Together with the initial validation of the AFS [15], this paper lends strong support for the utility of the AFS in both clinical and research settings. It addresses some of the limitations in current measures of child and adolescent functioning identified in two recent reviews [14, 16] supporting its use in policy and practice settings. The scale has evidence for change sensitivity and is a multi-dimensional measure developed specifically for the adolescent period. Further, the use of a 6-point rating scale better captures variability in responses, limiting floor and ceiling effects, therefore countering lower reliability seen with 2- or 3-point scales used in current measures [27].

To strengthen confidence in the use of the AFS as a multi-informant measure, a key focus of this study was to assess the equivalence of the AFS across the parent and adolescent versions. The AFS demonstrated configural and metric invariance across the parent and adolescent samples, indicating both formats are measuring similar aspects of adolescent functioning and have a similar factor structure. Scalar variance was not achieved, indicating that although the same constructs were being adequately assessed across formats, the scores themselves were not equivalent. For example, a high score on antisocial behaviour for adolescents was not equal to a high score for parents. This is common among many measures that assess perspectives of both parents and children [28, 29]. Developmental researchers argue that child development scales with multiple informants may be both useful and functionally equivalent even if they are not do achieve statistical scalar equivalence [30].

Study limitations include the use of convenience sampling, which resulted in low representation of fathers, different ethnicities, and parents and adolescents from socioeconomically disadvantaged backgrounds. Although these limitations are common in parenting research, even in large-scale international studies [31], they do limit generalisability. Perhaps more significantly, some of the under-represented groups (e.g., adolescents from culturally-diverse or disadvantaged families) are at greater risk of difficulties and are more likely to need mental health support. Thus, ongoing work is needed to establish clinical and community norms and to examine utility and validity within more diverse samples. Assessment of the discriminant validity of the AFS scores is also needed via comparison of the AFS to other measures that we would not be expected to be related to adolescent functioning. Further work is also needed with larger samples of adolescents to assess invariance across age groups within the developmental period of adolescence.

The AFS makes a significant contribution to the field as multiple-informant, multi-dimensional measure of adolescent functioning that includes positive development and is specifically tailored to adolescence rather than being an upward extension of a childhood measure. It is freely available, theory driven, change sensitive, internally consistent, and factorially sound. Moreover, there is evidence for the convergent and construct validity of the interpretations of test scores, and configural and metric invariance. Based on work to date, the AFS holds strong promise for use in clinical work and research as a measure of the assessment of positive and negative aspects of adolescent functioning from both parent and adolescent perspectives.

## Summary

The Adolescent Functioning Scale is a multi-dimensional measure of positive and negative domains of adolescent mental health and wellbeing designed for completion by adolescents and their parents. The current study provided further evidence for the validity of the 33-item Adolescent Functioning Scale (AFS) as a parent- and adolescent-report scale of adolescent adjustment comprising scales of positive development, oppositional behaviour, antisocial behaviour, and emotional problems subscales. Confirmatory factor analyses supported the 4-factor structure of the AFS in separate samples of parents (*N* = 542; 88% female) and adolescents aged 11–17 years (*N* = 303; 60% female). Analyses reduced the scale to produce a 28-item parent measure, and a 27-item adolescent measure. Evidence for convergent validity was provided through correlations with existing measures of adolescent functioning and parenting. The AFS demonstrated configural and metric invariance, but not scalar variance. The study provided support for the validity and reliability of the shorter version of the AFS for parents and adolescents. The AFS will have utility in research, intervention and applied contexts because of its brevity, strong psychometric properties, and capacity to be completed by parents and adolescents. Further, because it has been designed specifically for adolescents and includes a positive development scale, the AFS provides a brief, yet comprehensive, measure of mental health problems and developmental competencies with this age group.

### Supplementary Information

Below is the link to the electronic supplementary material.

Supplementary file1 (DOCX 44 kb)

## Data Availability

Data from this study is available through written request to the corresponding author. This study was not preregistered. The AFS is free for use and publicly available. The final parent-report and adolescent-report versions are included in the supplementary materials. Guidelines for use and information about translations can be found at https://pfsc.psychology.uq.edu.au/research/measures-library

## Footnotes

1 In Australia, adolescents typically start University when they are aged 17–18 years.

2 This is the Australian Equivalent to an Institutional Review Board (IRB).
