# Supplementary material for: Assessing Adolescent Functioning from Different Perspectives: Extending the Validation of the Adolescent Functioning Scale (AFS)
Source: Child Psychiatry Hum Dev. 2022 Sep 9;55(2):541–51. doi: 10.1007/s10578-022-01428-2 (PMC10891221; doi:10.1007/s10578-022-01428-2)
Supplement: Supplementary file 1 — Supplementary file1 (DOCX 44 kb) [file 10578_2022_1428_MOESM1_ESM.docx]

**Supplementary Material 1.** Parent-report version of the Adolescent Functioning Scale

**YOUR TEENAGER’S BEHAVIOUR AND EMOTIONS**

Below is a list of behaviours and emotions that relate to teenagers. Please read each statement below and rate from 0 (Not At All True) to 5 (True Most of the Time) how true the statement was of your teenager over the past four (4) weeks.

There are no right or wrong answers. Please do not spend too much time on any statement.

**EXAMPLE:**

| **My teenager:** | Not At all True |  |  |  |  | True Most of the Time |
| --- | --- | --- | --- | --- | --- | --- |
| 1. Refuses to get up for school | 0 | 1 | 2 | 3 | 4 | 5 |
| 1. Yells, shouts or screams | 0 | 1 | 2 | 3 | 4 | 5 |

| My teenager: | Not At all True |  |  |  |  | True Most of the Time |
| --- | --- | --- | --- | --- | --- | --- |
| 1. Constantly seeks reassurance | 0 | 1 | 2 | 3 | 4 | 5 |
| 1. Hurts me or others (e.g. hits, pushes, kicks) | 0 | 1 | 2 | 3 | 4 | 5 |
| 1. Loses their temper | 0 | 1 | 2 | 3 | 4 | 5 |
| 1. Gets involved in activities at school or in the community | 0 | 1 | 2 | 3 | 4 | 5 |
| 1. Talks about their views, ideas and needs appropriately | 0 | 1 | 2 | 3 | 4 | 5 |
| 1. Spends time with undesirable peers | 0 | 1 | 2 | 3 | 4 | 5 |
| 1. Is good at planning ahead for big tasks (e.g. assignments or exams) | 0 | 1 | 2 | 3 | 4 | 5 |
| 1. Puts themselves down | 0 | 1 | 2 | 3 | 4 | 5 |
| 1. Uses tobacco, drugs or alcohol | 0 | 1 | 2 | 3 | 4 | 5 |
| 1. Comes home late or misses their set curfew | 0 | 1 | 2 | 3 | 4 | 5 |
| 1. Seems unhappy or sad | 0 | 1 | 2 | 3 | 4 | 5 |
| 1. Rudely answers back to me | 0 | 1 | 2 | 3 | 4 | 5 |
| 1. Refuses to do jobs around the house when asked | 0 | 1 | 2 | 3 | 4 | 5 |
| 1. Tries hard at school/work/university | 0 | 1 | 2 | 3 | 4 | 5 |
| 1. Is irritable | 0 | 1 | 2 | 3 | 4 | 5 |
| 1. Doesn’t give up after a setback | 0 | 1 | 2 | 3 | 4 | 5 |

| My teenager: | Not At all True |  |  |  |  | True Most of the Time |
| --- | --- | --- | --- | --- | --- | --- |
| 1. Engages in risky or unhealthy activities | 0 | 1 | 2 | 3 | 4 | 5 |
| 1. Asks for advice about serious issues (e.g. drugs, sex, or relationships) | 0 | 1 | 2 | 3 | 4 | 5 |
| 1. Skips school, classes or work | 0 | 1 | 2 | 3 | 4 | 5 |
| 1. Does things for themselves | 0 | 1 | 2 | 3 | 4 | 5 |
| 1. Gets upset or angry when they don’t get their own way | 0 | 1 | 2 | 3 | 4 | 5 |
| 1. Whines or complains | 0 | 1 | 2 | 3 | 4 | 5 |
| 1. Thinks through consequences before acting | 0 | 1 | 2 | 3 | 4 | 5 |
| 1. Talks back or argues when asked to do something | 0 | 1 | 2 | 3 | 4 | 5 |
| 1. Has goals for the future | 0 | 1 | 2 | 3 | 4 | 5 |
| 1. Seems fearful and scared | 0 | 1 | 2 | 3 | 4 | 5 |
| 1. Worries | 0 | 1 | 2 | 3 | 4 | 5 |
| 1. Gets into trouble at school/college/work | 0 | 1 | 2 | 3 | 4 | 5 |

**Scoring**

Create a mean score for each subscale as follows:

Positive Development scale (9 items)

(4 + 5 + 7 + 14 + 16 + 18 + 20 + 23 + 25)/9

Oppositional Defiant Scale (8 items)

(2 + 3 + 12 + 13 + 15 + 21 + 22 + 24)/8

Antisocial Behaviour scale (6 items)

(6 + 9 + 10 + 17 + 19 + 28)/6

Emotional Difficulties scale (5 items)

(1 + 8 + 11 + 26 + 27)/5

**Supplementary Material 2.** Adolescent-report version of the Adolescent Functioning Scale

**YOUR TEENAGER’S BEHAVIOUR AND EMOTIONS**

Below is a list of behaviours and emotions that relate to teenagers. Please read each statement below and rate from 0 (Not At All True) to 5 (True Most of the Time) how true the statement was of your teenager over the past four (4) weeks.

There are no right or wrong answers. Please do not spend too much time on any statement.

**EXAMPLE:**

| **My teenager:** | Not At all True |  |  |  |  | True Most of the Time |
| --- | --- | --- | --- | --- | --- | --- |
| 1. Refuses to get up for school | 0 | 1 | 2 | 3 | 4 | 5 |
| 1. Yells, shouts or screams | 0 | 1 | 2 | 3 | 4 | 5 |

| My teenager: | Not At all True |  |  |  |  | True Most of the Time |
| --- | --- | --- | --- | --- | --- | --- |
| 1. Constantly seeks reassurance | 0 | 1 | 2 | 3 | 4 | 5 |
| 1. Hurts me or others (e.g. hits, pushes, kicks) | 0 | 1 | 2 | 3 | 4 | 5 |
| 1. Loses their temper | 0 | 1 | 2 | 3 | 4 | 5 |
| 1. Gets involved in activities at school or in the community | 0 | 1 | 2 | 3 | 4 | 5 |
| 1. Talks about their views, ideas and needs appropriately | 0 | 1 | 2 | 3 | 4 | 5 |
| 1. Spends time with undesirable peers | 0 | 1 | 2 | 3 | 4 | 5 |
| 1. Is good at planning ahead for big tasks (e.g. assignments or exams) | 0 | 1 | 2 | 3 | 4 | 5 |
| 1. Puts themselves down | 0 | 1 | 2 | 3 | 4 | 5 |
| 1. Uses tobacco, drugs or alcohol | 0 | 1 | 2 | 3 | 4 | 5 |
| 1. Comes home late or misses their set curfew | 0 | 1 | 2 | 3 | 4 | 5 |
| 1. Seems unhappy or sad | 0 | 1 | 2 | 3 | 4 | 5 |
| 1. Rudely answers back to me | 0 | 1 | 2 | 3 | 4 | 5 |
| 1. Refuses to do jobs around the house when asked | 0 | 1 | 2 | 3 | 4 | 5 |
| 1. Tries hard at school/work/university | 0 | 1 | 2 | 3 | 4 | 5 |
| 1. Is irritable | 0 | 1 | 2 | 3 | 4 | 5 |
| 1. Doesn’t give up after a setback | 0 | 1 | 2 | 3 | 4 | 5 |

| My teenager: | Not At all True |  |  |  |  | True Most of the Time |
| --- | --- | --- | --- | --- | --- | --- |
| 1. Engages in risky or unhealthy activities | 0 | 1 | 2 | 3 | 4 | 5 |
| 1. Asks for advice about serious issues (e.g. drugs, sex, or relationships) | 0 | 1 | 2 | 3 | 4 | 5 |
| 1. Skips school, classes or work | 0 | 1 | 2 | 3 | 4 | 5 |
| 1. Does things for themselves | 0 | 1 | 2 | 3 | 4 | 5 |
| 1. Gets upset or angry when they don’t get their own way | 0 | 1 | 2 | 3 | 4 | 5 |
| 1. Whines or complains | 0 | 1 | 2 | 3 | 4 | 5 |
| 1. Thinks through consequences before acting | 0 | 1 | 2 | 3 | 4 | 5 |
| 1. Talks back or argues when asked to do something | 0 | 1 | 2 | 3 | 4 | 5 |
| 1. Has goals for the future | 0 | 1 | 2 | 3 | 4 | 5 |
| 1. Seems fearful and scared | 0 | 1 | 2 | 3 | 4 | 5 |
| 1. Worries | 0 | 1 | 2 | 3 | 4 | 5 |

**Scoring**

Create a mean score for each subscale as follows:

Positive Development scale (9 items)

(4 + 5 + 7 + 14 + 16 + 18 + 20 + 23 + 25)/9

Oppositional Defiant Scale (8 items)

(2 + 3 + 12 + 13 + 15 + 21 + 22 + 24)/8

Antisocial Behaviour scale (5 items)

(6 + 9 + 10 + 17 + 19)/5

Emotional Difficulties scale (5 items)

(1 + 8 + 11 + 26 + 27)/5
